# The SUMO Protease SENP3 Orchestrates G2-M Transition and Spindle Assembly in Mouse Oocytes

**DOI:** 10.1038/srep15600

**Published:** 2015-10-23

**Authors:** Chun-Jie Huang, Di Wu, Faheem Ahmed Khan, Li-Jun Huo

**Affiliations:** 1 Key Laboratory of Agricultural Animal Genetics, Breeding and Reproduction, College of Animal Science and Technology, Huazhong Agricultural University, Wuhan, 430070, China

## Abstract

Oocyte meiosis is a transcription quiescence process and the cell-cycle progression is coordinated by multiple post-translational modifications, including SUMOylation. SENP3 an important deSUMOylation protease has been intensively studied in ribosome biogenesis and oxidative stress. However, the roles of SENP3 in cell-cycle regulation remain enigmatic, particularly for oocyte meiotic maturation. Here, we found that SENP3 co-localized with spindles during oocyte meiosis and silencing of SENP3 severely compromised the M phase entry (germinal vesicle breakdown, GVBD) and first polar body extrusion (PBI). The failure in polar body extrusion was due to the dysfunction of γ-tubulin that caused defective spindle morphogenesis. SENP3 depletion led to mislocalization and a substantial loss of Aurora A (an essential protein for MTOCs localization and spindle dynamics) while irregularly dispersed distribution of Bora (a binding partner and activator of Aurora A) in cytoplasm instead of concentrating at spindles. The SUMO-2/3 but not SUMO-1 conjugates were globally decreased by SENP3 RNAi. Additionally, the spindle assembly checkpoint remained functional upon SENP3 RNAi. Our findings renew the picture of SENP3 function by exploring its role in meiosis resumption, spindle assembly and following polar body emission during mouse oocyte meiotic maturation, which is potentially due to its proteolytic activity that facilitate SUMO-2/3 maturation.

The subfertility and chromosome mis-segregation in maternal aged oocytes has emerged as a serious globe health concern^1^,^2^,^3^. Oocyte meiosis is an error-prone process and mammalian oocytes can experience a protracted prophase I (germinal vesicle stage, GV) arrest even up to several decades^2^. Adding to this puzzle, the female is endowed with a definite pool of oocytes, which have to last her entire reproductive lifespan^4^. To ensure the fidelity of meiotic divisions to produce an euploid egg, chromosomes should biorient on the spindle under tension from dynamic spindle microtubules and align at the metaphase plate before allowing anaphase to proceed^5^,^6^,^7^. Given the striking role of spindle apparatus in chromosome segregation, therefore, any alternation in spindle physi-chemical properties might contribute to meiotic aneuploidy in mammalian oocytes^8^,^9^. Unlike mitotic cells, in which the bipolarity of spindle is conferred by a pair of centrosomes, in sharp contrast, the spindle of oocyte is acentrosomal and the spindle morphogenesis is orchestrated by a proteinaceous complex designated as Microtubule Organizing Centers (MTOCs)^10^,^11^ and additional microtubule nucleators^12^,^13^,^14^.

SUMOylation, one of the most dynamic post-translational modifications that exerts pivotal roles in dictating numerous cell-cycle events^15^,^16^ and this modification can be reversed by SUMO-specific enzymes (SENPs)^17^. Mammals expresses six SENPs (SENP1-3 and SENP5-7)^18^, those of which are liberated to varieties of roles including cohesion maintenance, spindle assembly and embryonic development^19^,^20^,^21^,^22^.

Among these SENPs, SENP3 tends to localize in nucleolus, and preferentialiy mediates SUMO-2/3 maturation and its removal from substrate^17^,^23^,^24^. Although its ribosome-based role have taken center stage^18^,^25^, SENP3 might have other functions involving cell-cycle regulation through removing SUMO-2/3 from mitotic Borealin^26^. Furthermore, Aurora B, CENP-E and BubR1 are all SUMO-2/3 targets in M phase^27^,^28^,^29^,^30^,^31^, leading us to postulate that SENP3 could be a unrecognized modulator of cell cycle progression. Significantly, however, it is not yet known whether SENP3 exerts any physiologically relevant during M phase.

Here, we detail SENP3 protein expression and localization during mouse oocyte meiosis. Importantly, we are trying to decipher how reduction in SENP3 native expression level affects meiotic progression. This entitles us to identify a novel function of SENP3 in cell-cycle timing and spindle morphogenesis that not only broadens SENP3’s function beyond ribosome biogenesis in mitotic cells but also defines SENP3 as a determinant of meiosis in mammal oocytes.

## Results

### Sub-localization and expression of SENP3 during mouse oocyte maturation

**Figure 1:**
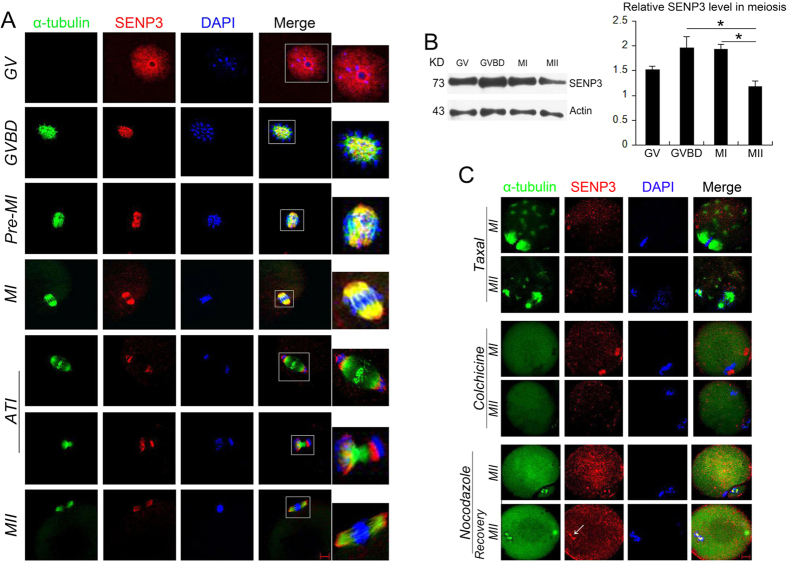
Sub-localization and expression of SENP3 during mouse oocyte maturation. (**A**) Subcellular localization of SENP3 detected by immunofluorescent staining. Oocytes at indicated stages were immunostained for SENP3 (red), microtubule (α-tubulin; green) and DNA (blue). (**B**) Expression of SENP3 during mouse oocyte meiotic maturation. Oocytes were collected after 0, 2, 8 or 14 h in culture, corresponding to GV, GVBD, MI and MII stage, respectively. The molecular mass of SENP3 is ~73 kDa and that of β-actin is 43 kDa. Normalized signal intensity of SENP3 was presented in the right panel. (**C**) Confocal images of SENP3 signal after treatment with different spindle drugs. Oocytes at indicated stage were double stained for SENP3 (red), α-tubulin (green) and DNA (blue). Magnificantion of the boxed regions showed SENP3 localized on spindles. Bar = 20 μm.

Our preliminary follicle immunofluorescent analysis revealed a nuclear signal of SENP3 in oocyte of preantral follicles (Fig. S1). Further study indicated that SENP3 predominantly localized in the nucleus with a bright signal external to nucleolus in GV-stage oocytes (Fig. 1A). Shortly after GVBD, SENP3 accumulated around the chromosome region and then co-localized with α-tubulin at the spindles in pre-MI and MI stage of oocytes; during chromosome separation at anaphase I-telophase I (ATI) stage, SENP3 localized between the separating chromosomes and the minus end of spindle microtubules. While in MII oocytes, SENP3 concentrated and re-localized at spindles. Moreover, western blot analysis showed that expression of SENP3 in oocytes was slightly increased from GV to GVBD stage and later declined in MI and MII (Fig. 1B).

The subcellular localization pattern of SENP3 dynamically associated with chromosomes and microtubules during oocyte meiotic maturation, which suggested an interaction between SENP3 and microtubules. The association between SENP3 with microtubules was further determined by employing spindle perturbation drugs, taxol, a microtubule-stabilizing chemical, and colchicine which could cause complete depolymerization of microtubules. The results were shown in Fig. 1C, taxol or colchicine treatment led to SENP3 dissociation from spindles and dispersed as cytoplasmic diffusing dots in MI and MII oocytes. Nocodazole, a reversible microtubule-depolymerizing agent, was also used, which could completely disassemble microtubules in MII oocytes and SENP3 signals were photocopied that in colchicine treated oocytes. However, when the nocodazole-treated MII oocytes were thoroughly washed and cultured in nocadazole-free medium to allow microtubule re-assembly, SENP3 signals were re-localized to the reforming spindle apparatus (Fig. 1C). Based on these evidence, SENP3 could be defined as a previously unknown microtubule-associated protein that potentially participates in oocyte meiosis regulation.

### Knockdown of SENP3 by siRNA compromises G2-M transition and first polar body extrusion

**Figure 2:**
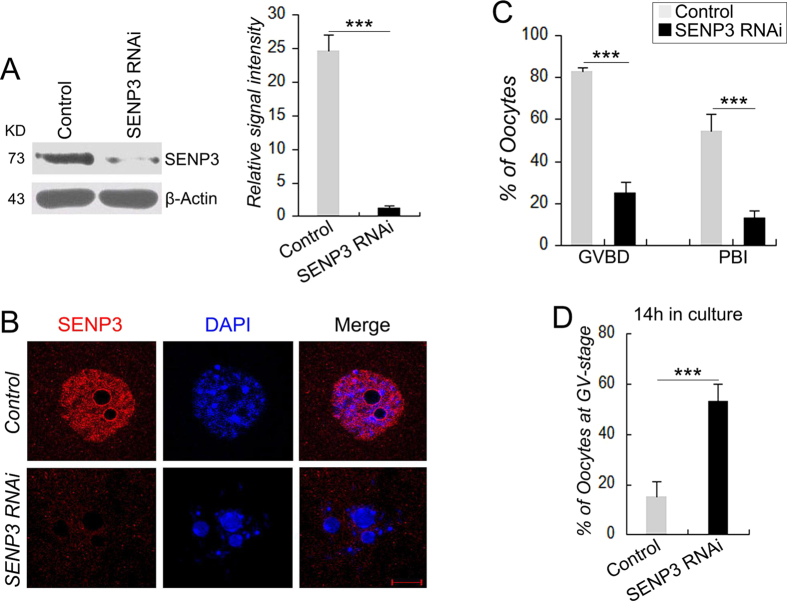
SENP3 RNAi compromises G2-M transition and first polar body extrusion. Oocytes injected with control or SENP3 siRNA were incubated in M2 medium containing 2.5 mM milrinone for 24 h and then released into milrinone-free M16 medium for continuously culture. (**A**) Immunoblot of SENP3 in control and SENP3 RNAi GV-stage oocytes. SENP3 relative expression level was normalized to values found in control oocytes (lower panel). (**B**) Knockdown efficiency of SENP3 protein was validated by immunostaining for SENP3 (red) and DNA (blue). (**C**) Characterization of GVBD and PBI rates of control (n = 147) and SENP3 RNAi oocytes (n = 143) following continuously observed at 2 h and 14 h, respectively. (**D**) Percentage of oocytes arrested at GV stage in control group (n = 168) and SENP3 RNAi group (n = 200) after 14 h in culture following release from milrinone. Data are presented as mean ± SEM. ***p < 0.001. Bar = 10 μm.

To evaluate the functional relevance of SENP3 during oocyte meiotic maturation, we interfered SENP3 expression by microinjecting a siRNA oligonucleotide against SENP3 into GV-stage oocytes. Western blot showed the successfully knockdown of SENP3 expression (~90%) by SENP3 RNAi after 24 h incubation in 2.5 μM milrinone (Fig. 2A). Immunofluorescent analysis further validated this knockdown efficacy as virtually no nuclear signals were detected after SENP3 RNAi (Fig. 2B). After SENP3 knockdown, oocytes were continuously cultured up to 14 h for maturation observation. We found the GVBD rate in SENP3 RNAi group was dramatically declined compared with that of in control group after 2 h in culture (25.2% vs 83.2%, p < 0.001, Fig. 2C). Furthermore, only a small proportion of oocytes extruded polar bodies in SENP3 RNAi group after 14 h in culture, compared to the control group (13.3% vs 60.1%, p < 0.001, Fig. 2C). Additionally, more than 50% of the oocytes in SENP3 RNAi group were still arrested at GV stage (n = 200), which was significantly higher than in the control group (n = 168, p < 0.001, Fig. 2D). Taken together, our results indicate that SENP3 knockdown severely interfere with both GVBD and PBI extrusion, implicating essential roles of SENP3 in oocyte meiotic maturation.

### SENP3 is Important for Pre-MI/MI Progression and Spindle Morphogenesis

**Figure 3:**
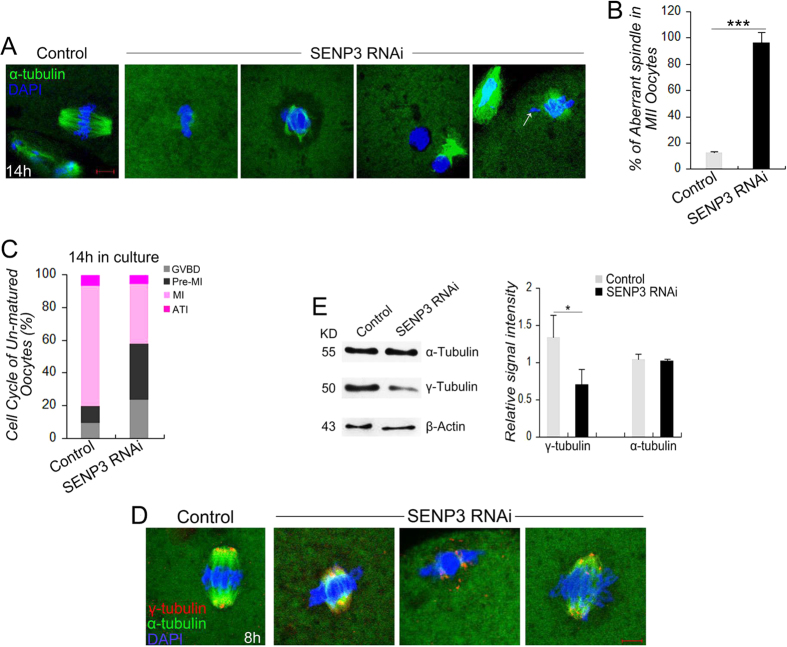
SENP3 is Important for PreMI/MI Progression and Spindle Morphogenesis. (**A**) Spindle morphology of control and SENP3 RNAi oocytes after 14 h in culture. Significantly, most SENP3 RNAi oocytes failed to eject the first polar body, showing various morphologically defective spindles. α-tubulin, green; DNA, blue. (**B**) The rate of aberrant spindles in MII oocytes in the control (n = 72) and SENP3 RNAi group (n = 12). (**C**) Graph showing the cell cycle of un-matured oocytes after 14 h in culture in control (n = 88) and SENP3 RNAi group (n = 104). (**D**) Confocal analysis of immunostained oocytes were used to determine the γ-tubulin localization in control and SENP3 RNAi oocytes post 8 h release form milrinone. γ-tubulin appears its typical spindle pole localization in control oocytes, while displayed various mislocalizations in SENP3 RNAi oocytes. α-tubulin, green; γ-tubulin, red; DNA, blue. (**E**) Immunoblot of α-tubulin and γ-tubulin in control and SENP3 RNAi oocytes. White arrowheads (**A**) mark the misaligned chromosomes in MII oocytes in SENP3 RNAi group. Data are presented as mean ± SEM. ***p < 0.001. Bar = 10 μm.

To deeply characterize the meiotic defects induced by SENP3 knockdown, we next examined the spindle assembly in oocytes after SENP3 knockdown. We found that after 14 h in culture, oocytes failed to extrude the polar bodies in SENP3 RNAi group showed faint signal of α-tubulin, dis-organized spindles, or microtubules only associating with one of the separating chromosomes, and even a few oocytes proceeded to MII stage also showed significantly higher level of aberrant spindles, compared with the normal barrel-shaped spindles in the control MII oocytes (Fig. 3A,B). Cell cycle analysis of those un-matured oocytes (post 14 h in culture) in SENP3 RNAi group revealed that majority of oocytes were arrested at GVBD or Pre-MI stage (Fig. 3C).

To further study the effect of SENP3 on spindle assembly, γ-tubulin, a well-known MTOC-associated protein which is essential for spindle morphogenesis, was examined. Co-localization of SENP3 with γ-tubulin in wide-type oocytes was studied. Our analysis found that the subcellular localization of SENP3 was closely associated with γ-tubulin during meiosis progression (Fig. S2). We then evaluated the localization of γ-tubulin in MI oocytes after SENP3 knockdown. In control MI oocytes, γ-tubulin localized at the spindle poles. In contrast, SENP3 RNAi markedly distorted the localization of γ-tubulin: γ-tubulin disassociated from the spindle poles and scattered around chromosomes or distributed along the spindles (Fig. 3D). Furthermore, the expression level of γ-tubulin but not α-tubulin, was significantly decreased in GV-stage oocytes after SENP3 RNAi (Fig. 3E). Consistent with this result, the immunostaining signal of acetylated α-tubulin, a marker for stable microtubules, displayed a similar pattern in both control and SENP3 RNAi groups (Fig. S3). Collectively, we could contend that γ-tubulin dysfunction (decreased expression level and disrupted localization) was involved in spindle morphogenetic defects induced by SENP3 knockdown.

### SENP3 knockdown disrupts bio-chemical properties of Aurora A and Bora

**Figure 4:**
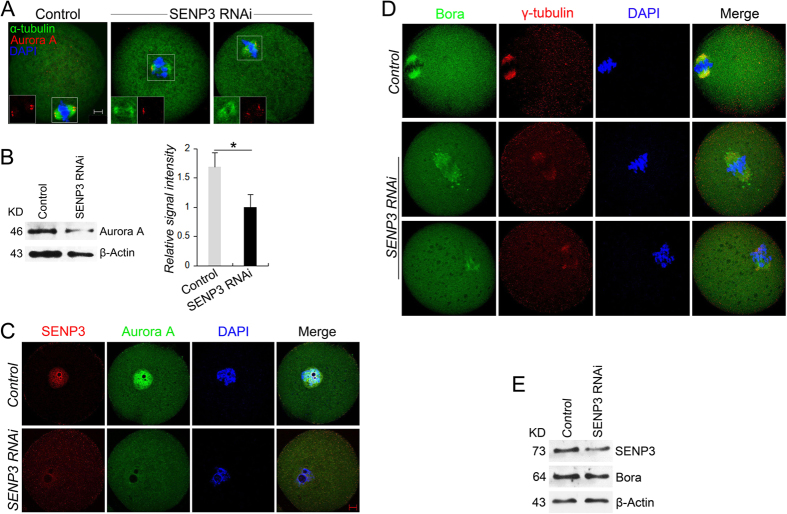
Localization and expression of Aurora A and Bora after SENP3 RNAi. Control or SENP3 siRNA injected oocytes were incubated in M2 medium containing 2.5 μM milrinone for 24 h before being continuously cultured in M16 medium. (**A**) In control MI oocyte after 8 h in culture, Aurora A localized at the spindle poles, while in SENP3 RNAi group, Aurora A signals disappeared or dispersed from the spindle poles. α-tubulin, green; Aurora A, red; DNA, blue. Bar = 10 μm. (**B**) Immunoblots of Aurora A in control and SENP3-depleted GV stage oocytes. Note that after SENP3 knockdown, Aurora A level was significantly decreased. Right panel is the normalized band intensity of Aurora A. (**C**) Confocal analysis of Aurora A in GV stage oocytes validated the reduction in Aurora A level after SENP3 RNAi. Note that in control group, Aurora A localized in GV and exhibited concentrated zones of signal around the nucleolar periphery. SENP3, red; Aurora A, green; DNA, blue. (**D**) Representative confocal images of control and SENP3 RNAi oocytes immunostained for Bora (green), γ-tubulin (red) and DNA (blue) after 8 h in culture. In the control group, Bora localized at the spindles. In the SENP3 RNAi group, localization of Bora was disrupted. (**E**) Immunoblots of Bora in control and SENP3 knockdown GV stage oocytes. *p < 0.05. Bar = 10 μm.

To uncover the potential mechanism of SENP3 on spindle morphogenesis, one of the SUMOylation target Aurora A, a well-recognized MTOC-associated protein that guarantees MTOCs localization by recruiting spindle assembly factors (SAFs), was examined. Significantly, Aurora A localized at the spindle poles in control MI oocytes, while in shape contrast, Aurora A signals were disappeared or dispersed around the spindles in SENP3-depleted oocytes (Fig. 4A). Furthermore, we revealed a significant loss of Aurora A expression in GV stage oocytes after SENP3 knockdown by western blot and confocal analyses (Fig. 4B,C).

Since Bora is a binding partner of Aurora A and is also involves in spindle formation and polar body extrusion, the localization and expression of Bora were studied after SENP3 RNAi. In control MI oocytes, Bora co-localized with the spindles. While in contrast, after SENP3 knockdown, Bora dispersed from the spindles and with irregular clusters around the chromosomes accompanied by defective γ-tubulin localization (Fig. 4D). However, the expression level of Bora in GV-stage oocytes was unaltered after SENP3 RNAi (Fig. 4E), indicating the diffusion of Bora into cytoplasm. Based on these evidence, we speculate that SENP3 might involve in microtubules assembly dictated by γ-tubulin through regulating the functions of Aurora A and Bora.

### SENP3 depletion alters the SUMO-2/3 not SUMO-1 mediated SUMOylation dynamics

**Figure 5:**
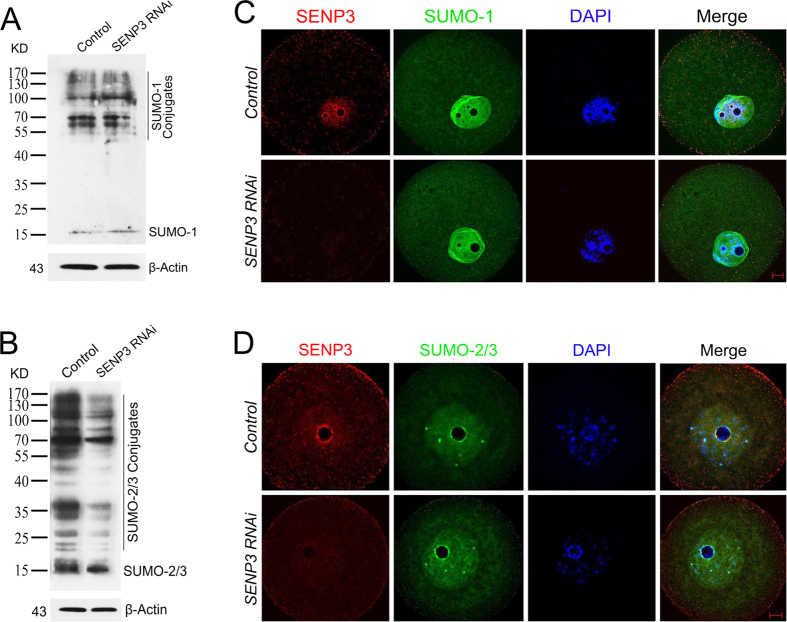
Effect of SENP3 RNAi on SUMOylation profile in mouse oocytes. In order to illustrate the preferentially activity of SENP3 to SUMO-1 or SUMO-2/3 mediated SUMOylation profile, quantitative immunoblots of SUMO-1 and its conjugates (**A**) as well as SUMO-2/3 and its conjugates (**B**) were measured in control and SENP3 RNAi GV oocytes. To study the effect of SENP3 RNAi on the localization of SUMO-1 and SUMO-2/3, control or SENP3 RNAi GV oocytes were immunostained for SENP3 and SUMO-1 (**C**) or SUMO-2/3 (**D**). Note that neither SUMO-1 nor SUMO-2/3 localization pattern was altered after SENP3 knockdown. SENP3, red; SUMO-1 and SUMO-2/3, green; DNA, blue. Bar = 10 μm.

SENP3 is known as a SUMO-specific protease that displays a high preference toward SUMO-2/3 over SUMO-1^32^. To study the effect of SENP3 knockdown on the dynamics of SUMO-1 and SUMO-2/3 conjugates, the GV-stage oocytes after SENP3 knockdown were collected for immunobloting analysis. The results showed that neither native SUMO-1 nor SUMO-1 conjugates was affected by SENP3 knockdown (Fig. 5A), nevertheless, SENP3 knockdown led to a global reduction in SUMO-2/3 conjugates (Fig. 5B). Notably, the localization of both SUMO-1 and SUMO-2/3 were unchanged after SENP3 RNAi (Fig. 5C,D). Taken together, our results suggest that SENP3 ablation initially hampers SUMO-2/3 maturation in mouse oocytes.

### SENP3 RNAi provokes the spindle assembly checkpoint

**Figure 6:**
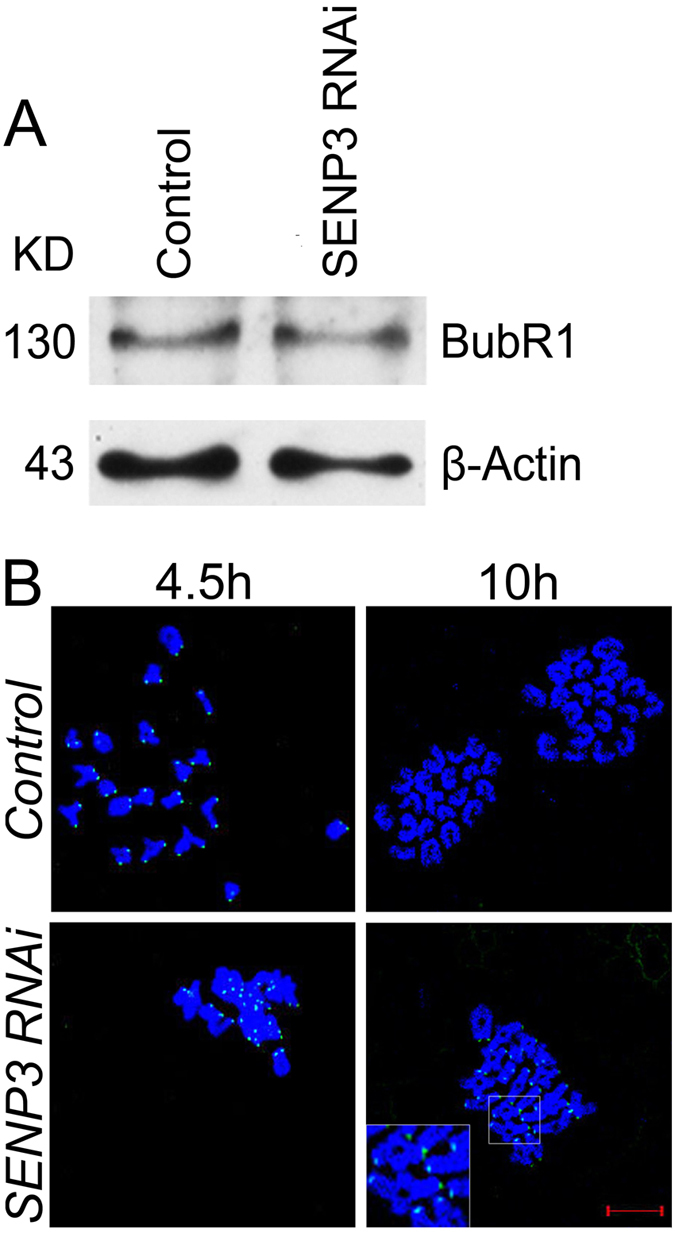
Spindle assembly checkpoint is functional after SENP3 depletion. Control or SENP3 siRNA injected GV oocytes were lysed for immunobloting of BubR1 (**A**) or cultured in M16 medium for 4.5 h and 10 h, corresponding to PreMI and AI stage, followed by chromosome spreading of BubR1 (**B**). BubR1, green; Blue, DNA. Bar = 10 μm.

We observed a severely PBE defect was induced by SENP3 knockdown (Fig. 2C). The SENP3-knockdown oocytes failed to complete meiosis I division motivated us to determine the activity of **spindle assembly checkpoint (**SAC). We analyzed BubR1 in oocytes, which is a principal component of SAC and also a target of SUMOylation^19^. As predicted, the BubR1 expression level was indistinguishable in both groups (Fig. 6A). We additionally assayed the SAC activity in control and SENP3-depleted oocytes after 4, 5 h and 10 h in culture, the time point presenting oocytes proceed to pre-MI and anaphase I, respectively. The signals of BubR1 localized at kinetochores were comparable in both groups at pre-MI stage. When oocytes proceed to anaphase I, the homologous chromosomes were segregated and kinetochore-localization of BubR1 was disappeared in control oocytes. However, in SENP3-depleted oocytes, the homologous chromosomes were still held together by cohesion and BubR1 signals were persisted at kinetochores (Fig. 6B), further confirming that SAC was functional after SENP3 depletion.

## Discussion

Oocyte meiosis is an error-prone process and understanding how meiotic resumption occurs and microtubules rearrange into meiotic bipolar spindles remain challenges in oocyte biology^2^,^8^. To our knowledge, this is the first report deciphering that SENP3 is essential for meiotic resumption and spindle assembly during mouse oocyte maturation. The results showed that knockdown of SENP3 severely compromised PBI ejection by disrupting γ-tubulin organization and spindle morphogenesis in mouse oocytes. Further study revealed that disruption of γ-tubulin might be due to the dsyfunction of Aurora A and Bora, which is presumably caused by the attenuated SUMO-2/3-ylation dynamic after SENP3 RNAi.

In mitotic cells, SENPs exert distinct roles in cell-cycle regulation presumably due to their different intracellular distribution and differential activities to SUMO isoforms^22^,^33^. In our study, the expression profile of SENP3 during oocytes maturation coincides with the previous proteomic study^34^. Immunofluorecent analysis revealed the signal of SENP3 is external to nucleolus at GV-stage oocyte, which is inconsistent with the established intranucleolus localization of SENP3 in mitotic cells^24^. A small fraction of SENP3 RNAi oocytes were preceded to MII stage, because probably, we could not knockout all SENP3 expressions, which might be sufficient to support maturation but not proper spindle assembly. Similarly, despite GVBD was only measured at a single time point (2 h post release from milrinone), more than 50% of oocytes are still at GV-stage even after 14 h in culture, supporting that GVBD is indeed impaired after SENP3 RNAi. Thus, we propose that SENP3 is crucial for meiotic progression and the precipitous decline in PBI extrusion is ascribed to both GVBD and spindle assembly defects.

The spindle morphogenesis in mammal oocyte is dictated by MTOCs and additional MT nucleators^10^,^35^. Here, the reduction in γ-tubulin level after SENP3 knockdown might be due to ubiquitination mediated proteasomal degradation of γ-tubulin^36^. Furthermore, native Aurora A abundance was significantly declined in SENP3 RNAi group, indicative of Aurora A loss is causally linked to the MTOCs mislocalization and aberrant spindle formation. Distribution of its activator Bora is also disrupted in this study. These findings are in accordance with previous studies showing that Bora and Aurora A are required for MTOCs localization and spindle morphology^37^,^38^. Aurora A is required for recruiting and phosphorylation of many proteins such as kinetochore-specific protein CENP-A^39^,^40^. Perturbation of CENP-A phosphorylation leads to chromosomal misalignment at metaphase plate in mammalian cells^41^,^42^. Interestingly, chromosomes were frequently misaligned in our study, thus, we could not rule out the possibility that CENP-A phosphorylation is inhibited due to Aurora A reduction. Intriguing, Aurora A and Bora are destructed by hCdc14A-mediated phosphorylation and Plk1-dependent proteasome proteolysis^43^, therefore, in our study how Bora expression remains unchanged while Aurora A level is reduced deserve future investigation.

Another conspicuous phenotype as a result of SENP3 knockdown is the damaged G2-M transition. The meiosis resumption of oocytes just resembles the mitotic entry in somatic cells^44^. Mitotic entry is a sophisticated process and is initiated by the activation of the Cyclin B-CDK1 complex in most species^45^,^46^,^47^. Aurora A participates in mitotic entry by facilitating centrosomal recruitment of Cyclin B (CCNB) and phosphorylate CDC25B^48^,^49^. Significantly, however, further studies experimentally challenged the role of Aurora A in mouse oocyte meiosis resumption^50^,^51^, making the impaired GVBD after SENP3 RNAi appears unlikely to be explained by Aurora A down-regulation. Intriguing, we detected two bands of cyclin B1, the upper band of which is supposed to be Cyclin B1 phosphorylated status and its intensity was slightly decreased after SENP3 knockdown. Alternatively, the two bands of cyclin B1 are due to the cross-activity of antibody to other related target (Fig. S3). Hec-1 a subunit of the Ndc80 complex that exerts a pivotal kinetochore-based role was recently found to be required for stabilizing cyclin B2, which is a newly recognized determinant of G2-M transition in mammal oocytes^11^. Importantly, SENP3 was reported to activate p53 by binding of Mdm2 to hamper Mdm2-mediated p53 proteolysis^52^, thus, whether SENP3 affects GVBD through stabilizing one specific cyclin becomes an attractive question.

SUMOylation is involved in myriad cellular events^15^,^16^, SUMO-1 physically localizes to spindle in both mitotic and meiotic cells^19^,^53^. Not surprising, several microtubule-associated proteins are targets of SUMO-1, such as Aurora A, RanGAP1 and NuMA and this modification is required for their normal spindle localization and functions^16^,^27^,^53^. In contrast, SUMO-2/3 are more concentrated at centromeres, and thus, proteins located at centromeres and kinetochores are often modified by SUMO-2/3, including BubR1, Topoisomerase IIa and Aurora B^15^,^54^. In line with previous studies^17^,^32^, we found SENP3 knockdown exerts no effect on SUMO-1-ylation dynamic, however, SUMO-2/3 conjugates are globally attenuated. SUMOylation is likely to simultaneously modified multiple targets to cause a significant phenotype^15^, therefore, it is plausible that the aberrant spindle formation and chromosome dynamics in SENP3 deficiency oocytes could be partially explained by the unbalanced SUMO-2/3-ylation of some of the aforementioned or unknown targets. Alternatively, SENP3 may serve as a scaffold, facilitating its chaperone binding to MTs just like DDA3^55^. Note that, neither kinetochore-localization nor expression of BubR1 is affected after SENP3 RNAi, implying that SAC is provoked by the defective spindle morphogenesis in SENP3 RNAi oocytes to delay the anaphase I onset. In contrast to control oocytes that BubR1 signals were disappeared from kinetochores after 10 h in culture, the BubR1 signals were were persisted at kinetochores in SENP3-depleted oocytes, further confirming the functional of SAC. There is still possibility that the dissociation of BubR1 from kinetochores is attenuated after SENP3 RNAi. Further identification of SENP3 functional partners during M phase would be a pressing need for better elucidating its role in oocyte meiosis.

Overall, by extending SENP3’s functions beyond previously reported, our study makes SENP3 a critical node for integrating M phase entry with spindle assembly in mouse oocytes, which presumbly owing to its processivity that facilitate SUMO-2/3 maturation.

## Methods and Materials

### Antibodies and reagents

Rabbit anti-SENP3 monoclonal antibody (Cat# 5591) and rabbit anti-SUMO-1 ployclonal antibody (Cat# 4930) were purchased from CST (Danvers, MA); sheep anti-BubR1 ployclonal antibody (Cat# 28193), mouse anti-Aurora A monoclonal antibody (Cat# 28193) and rabbit anti-SUMO-2/3 monoclonal antibody (Cat# 109005) were purchased from Abcam (Cambridge, UK); mouse monoclonal anti-α-tubulin-FITC antibody (Cat# F2168) was obtained from Sigma (St Louis, MO). FITC- conjugated donkey anti-sheep IgG (H+L) was produced by Jackson ImmunoResearch Laboratories, Inc.

Spindle pertubation drugs taxol, colchicine and nocodazole were 5 mM, 10 mg/ml and 10 mg/ml in DMSO stock (−20 °C), respectively, were purchased from Sigma-Aldrich Co. All other reagents were purchased from Sigma Aldrich unless specifically stated otherwise.

### Animals and Ethics statement

Kunming strain (KM) mice were obtained from local Central Animal Laboratory and were bred at the experimental animal centre of Huazhong Agricultural University under a 12 h light/dark cycle with water and food ad libitum. This study was approved by the Ethical Committee of the Hubei Research Center of Experimental Animals (Approval ID: SCXK (Hubei) 20080005). All experimental protocols were conducted in accordance with the guidelines of the Committee of Animal Research Institute, HuaZhong Agricultural University, China.

### Oocyte collection, culture and drug treatment

Ovaries were isolated from 3–4 week-old KM mice sacrificed by cervical dislocation and fully grown, GV (germinal vesicle)-intact oocytes were collected in pre-warmed (37 °C) M2 medium supplemented with 2.5 μM milrinone to arrest the oocytes at GV-stage. To induce meiotic maturation, oocytes were washed out of milrinone and cultured in M16 medium for 0, 2, 4.5, 8, 9.5 and 14 h, corresponding to GV stage, germinal vesicle breakdown (GVBD) stage, pre-metaphase I (Pre-MI), metaphase I (MI), anaphase I (AI) and metaphase II (MII), respectively. After specific periods of culture, oocytes were collected for GVBD or PBI (MII) observation, drug treatment, microinjection, western blotting or immunofluorescent analysis.

For drug treatment, wide-type MI or MII stage oocytes were incubated in M16 medium containing 10 μM of taxol for 45 min, 10 μg/ml of colchicine for 1 h, or 20 μg/ml of nocodazole for 15 min, respectively, followed by immunostaining of SENP3 and α-tubulin. For microtubule re-assembly, after incubation with nocodazole for 15 min, oocytes were then washed thoroughly and recovered in fresh M16 medium for 30 min, and collected for immunofluorescent analysis. All control oocytes were cultured in M16 medium containing the same concentration of DMSO.

### Immunofluorescence and confocal microscopy

Specific stage of oocytes were briefly washed through PHEM solution (60 mM PIPES at pH 6.9, 25 mM HEPES, 10 mM EGTA, 2 mM MgCl_2_.7H_2_O) and fixed using 4% paraformaldehyde in PHEM containing 0.5% Triton X-100 for 30 min. Oocytes were then blocked in PBS containing 3% BSA and 0.05% Tween-20 for 1 h at room temperature and incubated with proper primary antibodies overnight at 4 °C or 2 h at 37 °C. After washing in PBS containing 0.05% Tween-20 for 3 times and 10 min each, oocytes were incubated with the corresponding secondary antibodies for 2 h at 37 °C. For double-staining, after secondary antibody incubation, oocytes were blocked again in blocking solution for 1 h at room temperature, and then incubated with the other primary antibodies, oocytes were processed with the secondary antibodies corresponding to the second primary antibodies. DNA was labelled in PBS containing 1 μg/ml of DAPI for 10 min at room temperature. Finally, oocytes were mounted on glass slides with DABCO and examined with a confocal laser scanning microscope (Zeiss LSM 510 META, Carl Zeiss Imaging, Germany) equipped with a Plan-Apochromat 63×/1.4 oil DIC objective. Confocal images were processed using Zeiss LSM Image Browser software and Adobe Photoshop (Adobe Systems Inc., San Jose, CA). For the negative control, non-immunized rabbit or mouse IgG were used to replace the primary antibodies.

For immunolabelling, the following primary antibodies and dilutions were used: rabbit anti-SENP3 antibody (1:100), rabbit anti-Bora antibody (Abcam; 1:100), rabbit anti-Aurora A (1:180), rabbit anti-SUMO-1 (1:100), rabbit anti-SUMO-2/3 (1:100), mouse anti-γ-tubulin antibody (Boster, China; 1:50), rabbit anti-Ac-α-tubulin (CST; 1:100), rabbit anti-Cyclin B1 antibody (Affinity; 1:100) or FITC-labelled mouse anti-α-tubulin (1:100). FITC- or Cy3-labelled goat anti-rabbit or goat anti-mouse (Boster; 1:100) antibodies were used as the secondary antibodies.

### Chromosome spreading

For BubR1 detecting, chromosome spreading was performed as described previously^56^. Briefly, the zona pellucida was removed prior to fixation by briefly exposure to 1% pronase in M16 medium and oocytes were then fixed on slides with 1% paraformaldehyde in distilled H2O (pH 9.2) containing 0.15% Triton X-100 and 3 mM dithiothreitol. The air-dried slides were washed and blocked with PBS containing 2% BSA, followed by incubating with sheep anti-BubR1 antibody (1:100) and FITC-conjugated donkey anti-sheep antibody (1:100) and DNA was visualized by DAPI staining. Finally, the slides were mounted and examined with the confocal laser scanning microscope.

### Knockdown of SENP3 by siRNA microinjection

For microinjection, GV-stage oocytes were collected in M2 medium containing 2.5 μM milrinone, and 5–10 pL of 30 μM control siRNA (sc-37007; Santa Cruz, CA) or SENP3 siRNA (sc-45178; Santa Cruz, CA) was injected into the cytoplasm of oocyte. Following microinjection, oocytes were cultured in M2 medium supplemented with 2.5 μM milrinone for 24 h to achieve SENP3 knockdown. After that, oocytes were directly collected for western blotting or immunostaining or thoroughly washed out of milrinone and released into M16 medium for meiotic maturation or other experiments.

### Immunoblotting

About 200 oocytes each group were briefly washed in PBS and then lysed in 2 × SDS sample buffer and stored at −80 °C until use. For blotting, samples were heated at 100 °C for 5 min and then placed on ice for 5 min. The proteins were separated by SDS-PAGE and electrically transferred to PVDF membranes (Immobilon-P; Millipore). The membranes were blocked in 5% BSA in TBS (25 mM Tris, 150 mM NaCl, pH 8.0) containing 0.01% Tween-20 (TBST) for 1 h and then incubated overnight at 4 °C with the primary antibodies, respectively, including rabbit monoclonal anti-SENP3 (1:1000), rabbit monoclonal anti-α-tubulin (1:1000), mouse polyclonal anti-γ-tubulin (1:200), rabbit polyclonal anti-Aurora A (1:1000), rabbit monoclonal anti-Bora (1:1000), rabbit polyclonal anti-SUMO-1 (1:1000), rabbit monoclonal anti-SUMO-2/3 (1:1000) and sheep polyclonal anti-BubR1 (1:1000). After washing with TBST, secondary antibodies were used as anti-mouse or anti-rabbit HRP conjugates (1:3000; Boster), the immunoblot bands were visualized with ECL kit and read using chemiluminescence system (Thermo Scientific). For semi-quantitative analysis, the immunoblot protein bands were assayed by the ChemiDoc XRS Imaging System (Bio-Rad). Beta-actin (1:2000; Santa Cruz, CA) was served as a loading control. The relative signal intensity was assessed by ImageJ software (NIH, USA). For the negative control, the non-immunized rabbit or mouse IgG were used to replace the primary antibodies.

### Statistical Analysis

Data were presented as mean ± SEM from three repeated experiments and analyzed by paired-samples *t*-test using SPSS software (SPSS Inc, Chicago, IL) with p < 0.05 was considered to be statistically significant. Different superscripts indicate the statistical difference. The numbers of oocytes collected were mentioned as (n) in parentheses.

## Additional Information

**How to cite this article**: Huang, C.-J. *et al.* The SUMO Protease SENP3 Orchestrates G2-M Transition and Spindle Assembly in Mouse Oocytes. *Sci. Rep.* **5**, 15600; doi: 10.1038/srep15600 (2015).

## Supplementary Material

Supplementary Information
