# Supplementary material for: The SUMO Protease SENP3 Orchestrates G2-M Transition and Spindle Assembly in Mouse Oocytes
Source: Sci Rep. 2015 Oct 23;5:15600. doi: 10.1038/srep15600 (PMC4616058; doi:10.1038/srep15600)

**The SUMO Protease SENP3 Orchestrates G2-M Transition and Spindle Assembly in Mouse Oocytes**

Chun-Jie Huang, Di Wu, Faheem Ahmed Khan & Li-Jun Huo^*^

Key Laboratory of Agricultural Animal Genetics, Breeding and Reproduction, College of Animal Science and Technology, Huazhong Agricultural University, Wuhan, China

^*^Correspondence to: Li-Jun Huo; Fax: 86-27-87288376; Email: [lijunhuo@yahoo.com](mailto:lijunhuo@yahoo.com)

**Supplementary information**

**Figure S1. Localization of SENP3 in *in vivo* preantral follicles**

Various developmental stages of preantral follicles were isolated and prepared for immunostained for SENP3 and DNA. The localization pattern of SENP3 was constant during preantral follicles development with a bright signal external to nucleolus. SENP3, red; DNA, blue. Bar = 20 μm.

**Figure S2. Co-localization of SENP3 and γ-tubulin during mouse oocyte maturation.**

Oocytes at specific stages were co-immunostained with SENP3 (red), γ-tubulin (green) and DNA (blue). Bar = 20 μm.

**Figure S3. Effect of SENP3 RNAi on microtubulin stability at GV-stage oocytes.**

Optimized confocal Z section of acetylated α-tubulin at GV oocytes. Note that the microtubule acetylation level is indistinguishable in control and SENP3 RNAi group. Ac-α-tubulin, red; DNA, blue. Bar = 10 μm.

**Figure S4. Expression level of cyclin B1 after SENP3 RNAi**

GV-stage oocytes of control or SENP3 RNAi group were maintained in milrinone for 24 h and then lysed for immunobloting of cyclin B1. Left panel is the immunoblot bands of cyclin B1 while the right panel represents the normalized intensity of bands corresponding to the left panel.

**Figure S1. Localization of SENP3 in *in vivo* preantral follicles**

**
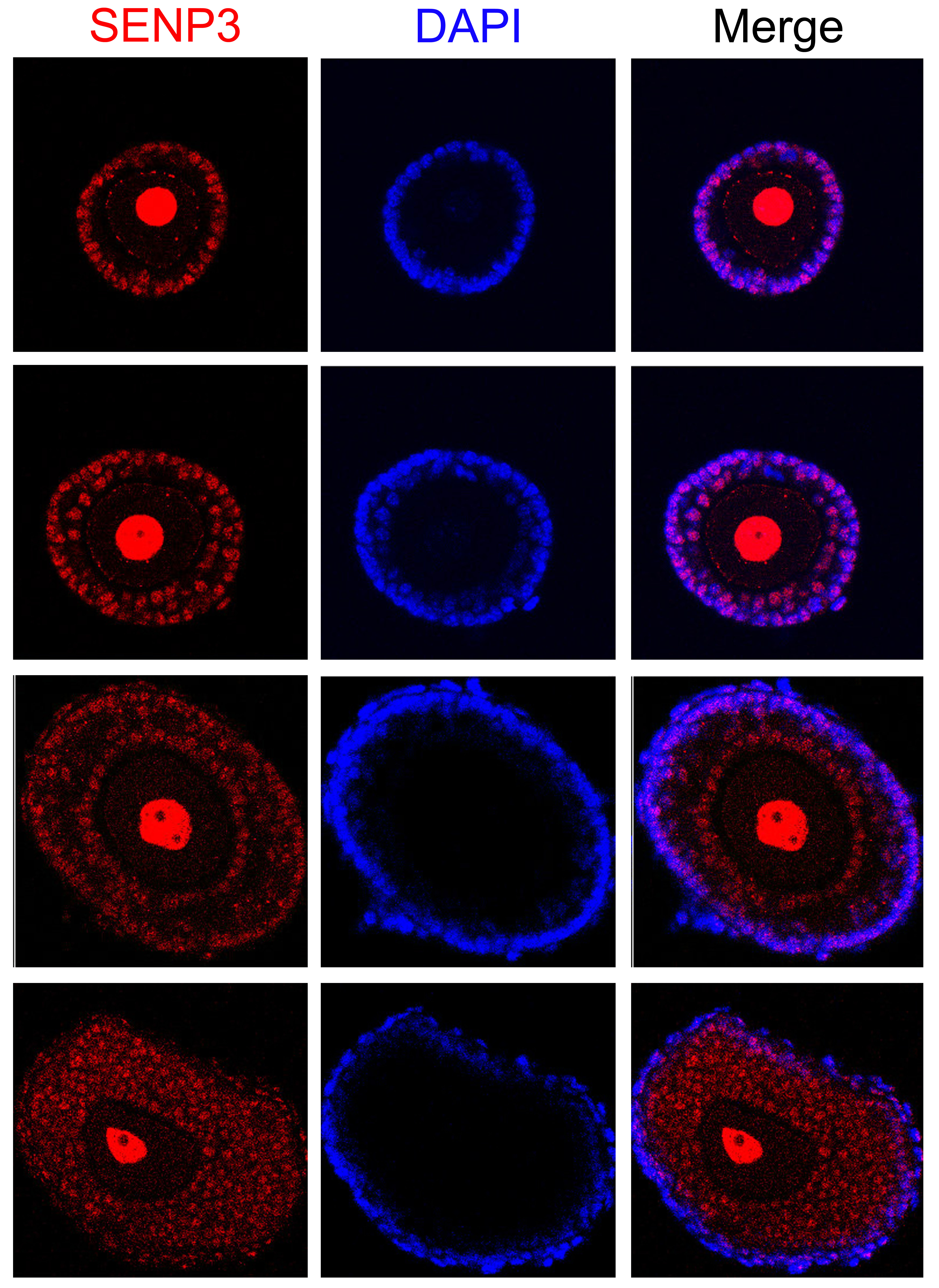
**
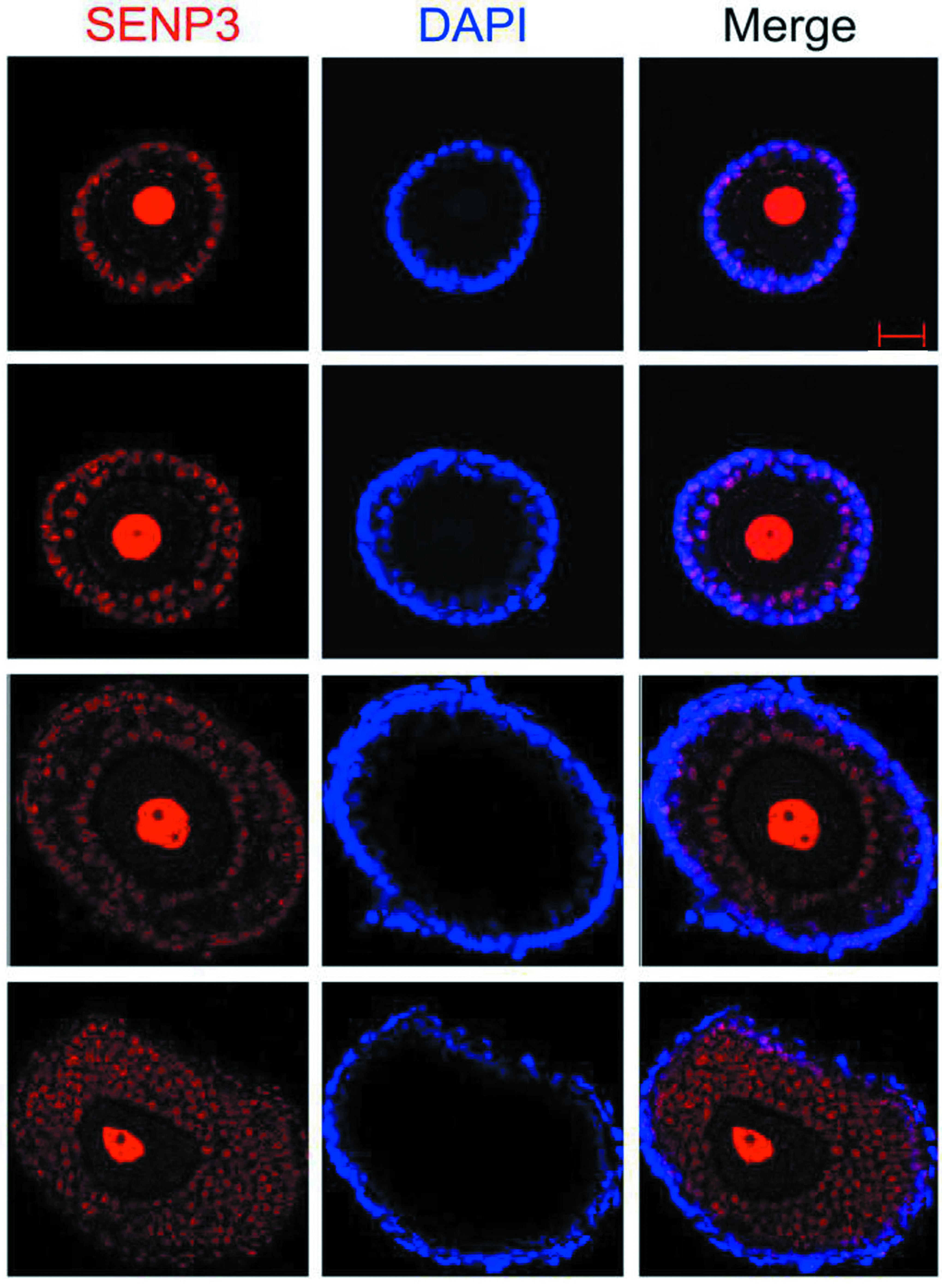


**Figure S2. Co-localization of SENP3 and γ-tubulin during mouse oocyte maturation.**


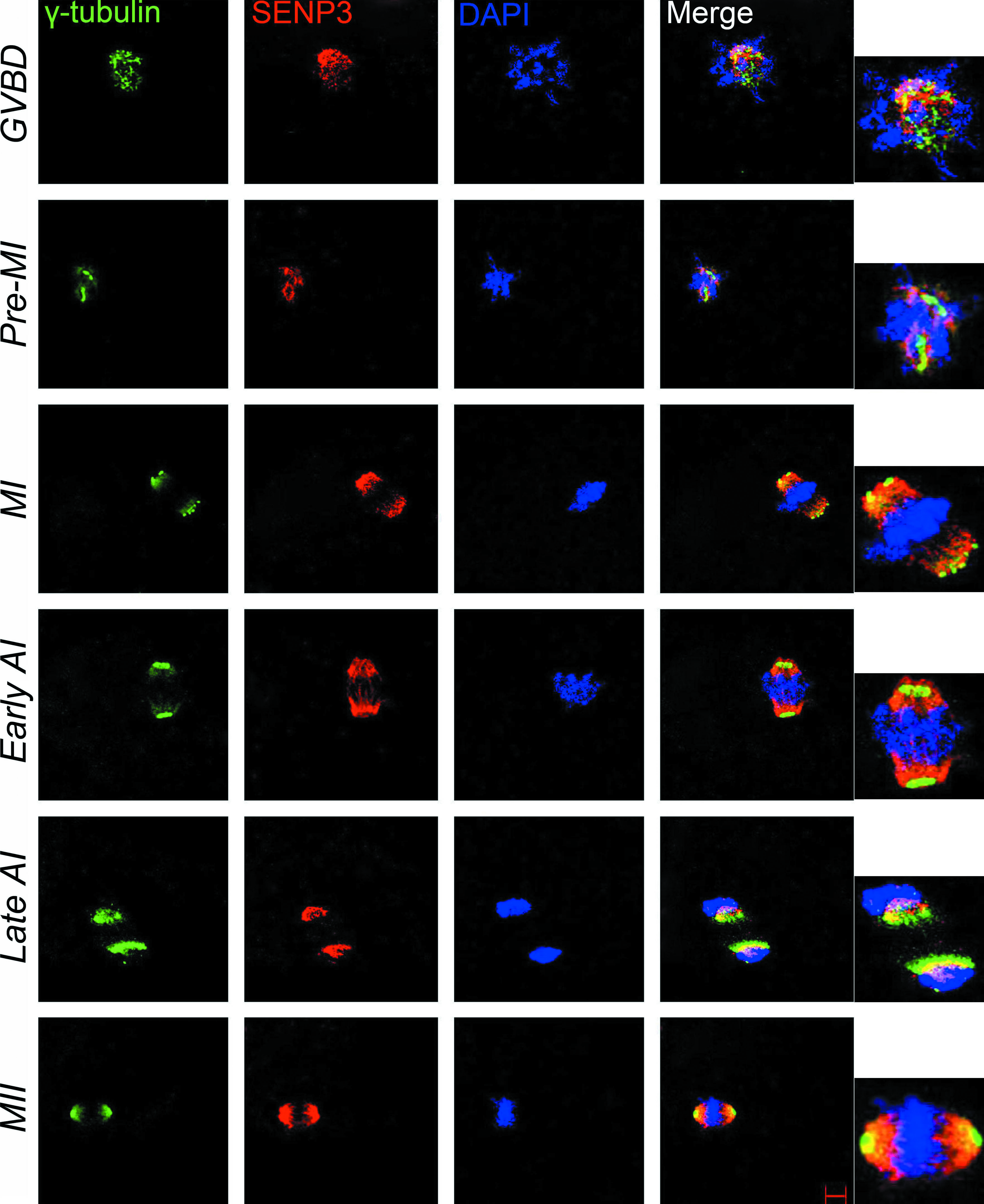


**Figure S3. Effect of SENP3 RNAi on microtubulin stability at GV-stage oocytes.**


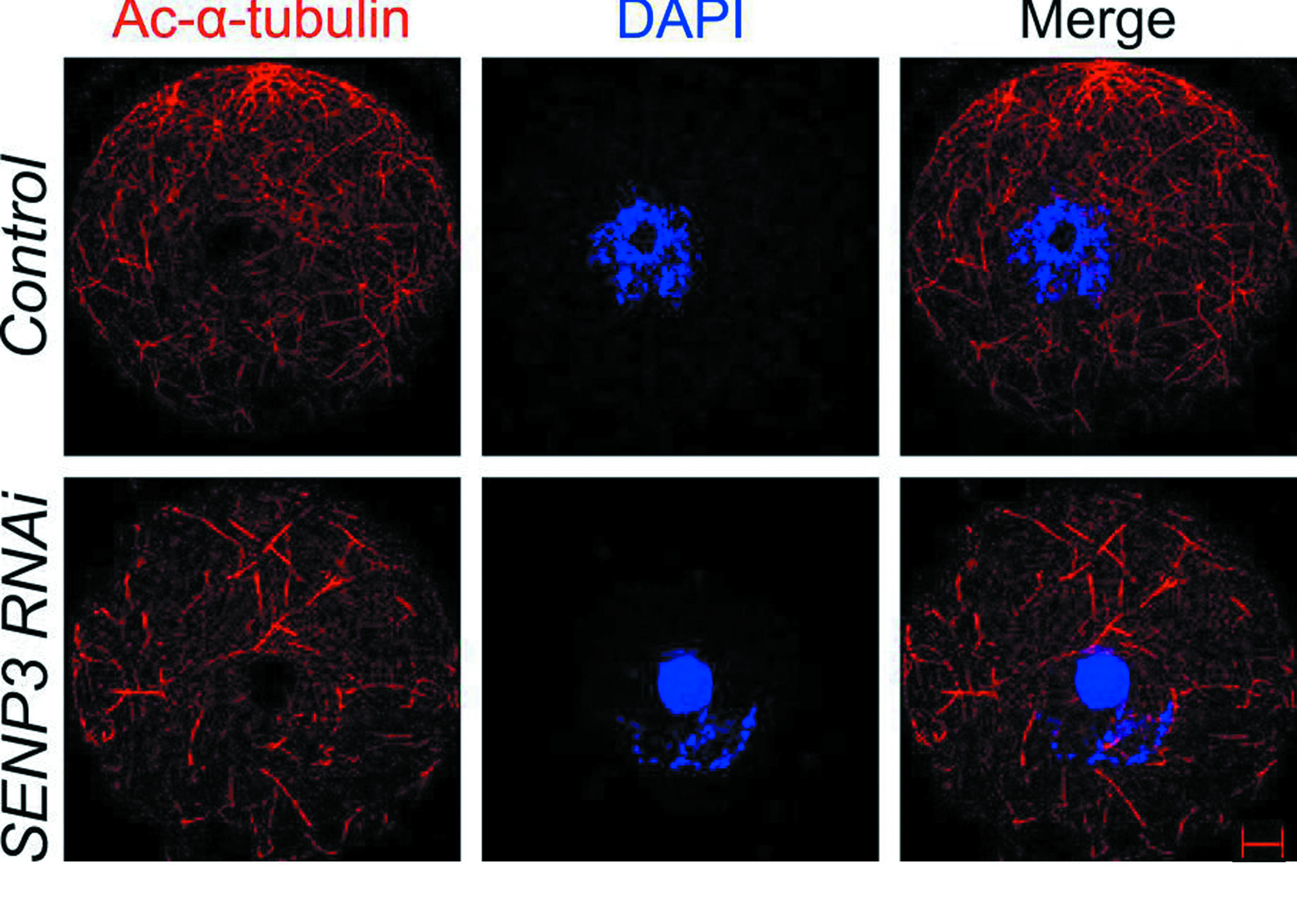


**Figure S4. Expression level of cyclin B1 after SENP3 RNAi**


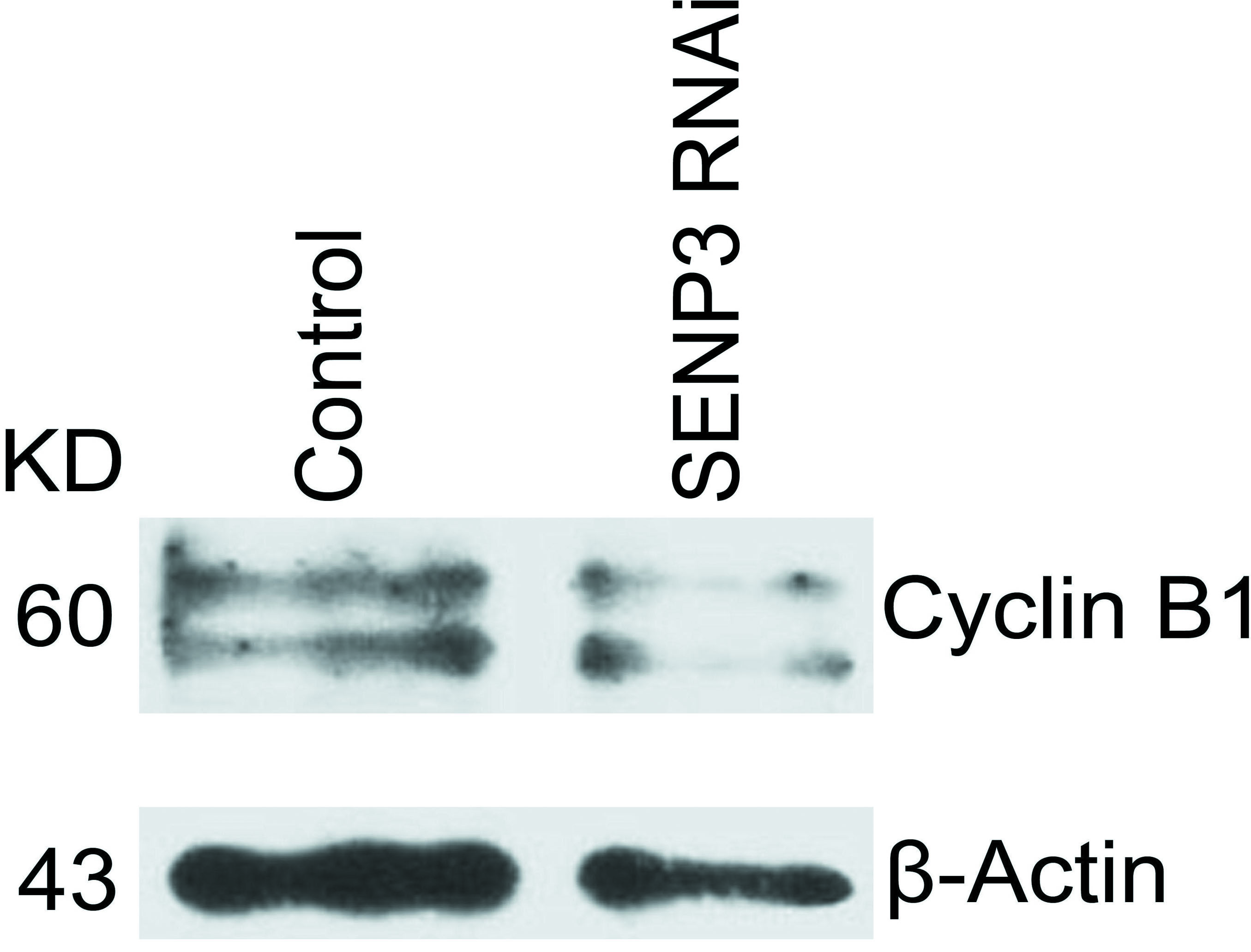

Supplement: Supplementary Information [file srep15600-s1.doc]
